# Plasma phospho-tau in Alzheimer’s disease: towards diagnostic and therapeutic trial applications

**DOI:** 10.1186/s13024-023-00605-8

**Published:** 2023-03-16

**Authors:** Fernando Gonzalez-Ortiz, Przemysław R. Kac, Wagner S. Brum, Henrik Zetterberg, Kaj Blennow, Thomas K. Karikari

**Affiliations:** 1 Department of Psychiatry and Neurochemistry, Institute of Neuroscience and Physiology, The Sahlgrenska Academy, University of Gothenburg, Gothenburg, Sweden; 2 Clinical Neurochemistry Laboratory, Sahlgrenska University Hospital, Mölndal, Sweden; 3 Graduate Program in Biological Sciences: Biochemistry, Universidade Federal Do Rio Grande Do Sul (UFRGS), Porto Alegre, Brazil; 4 Department of Neurodegenerative Disease, UCL Institute of Neurology, London, UK; 5 UK Dementia Research Institute at UCL, London, UK; 6 Hong Kong Center for Neurodegenerative Diseases, Hong Kong, China; 7 Department of Psychiatry, University of Pittsburgh, Pittsburgh, PA USA

**Keywords:** Alzheimer, s disease, Phosphorylated tau, Blood biomarker, Plasma p-tau, Dementia

## Abstract

As the leading cause of dementia, Alzheimer's disease (AD) is a major burden on affected individuals, their families and caregivers, and healthcare systems. Although AD can be identified and diagnosed by cerebrospinal fluid or neuroimaging biomarkers that concord with neuropathological evidence and clinical symptoms, challenges regarding practicality and accessibility hinder their widespread availability and implementation. Consequently, many people with suspected cognitive impairment due to AD do not receive a biomarker-supported diagnosis. Blood biomarkers have the capacity to help expand access to AD diagnostics worldwide. One such promising biomarker is plasma phosphorylated tau (p-tau), which has demonstrated specificity to AD versus non-AD neurodegenerative diseases, and will be extremely important to inform on clinical diagnosis and eligibility for therapies that have recently been approved. This review provides an update on the diagnostic and prognostic performances of plasma p-tau181, p-tau217 and p-tau231, and their associations with in vivo and autopsy-verified diagnosis and pathological hallmarks. Additionally, we discuss potential applications and unanswered questions of plasma p-tau for therapeutic trials, given their recent addition to the biomarker toolbox for participant screening, recruitment and during-trial monitoring. Outstanding questions include assay standardization, threshold generation and biomarker verification in diverse cohorts reflective of the wider community attending memory clinics and included in clinical trials.

## Background

As the leading cause of dementia worldwide, Alzheimer’s disease (AD) continues to present urgent strains on clinical care, public health efforts, palliative care and family systems [1]. Although the ultimate confirmation of AD pathology is by autopsy examination of brain tissue for extracellular amyloid plaques made of amyloid-beta (Aβ) peptides and intraneuronal neurofibrillary tangles (NFTs) containing phosphorylated tau (p-tau) forms [2, 3], in vivo diagnosis is presently achieved by using either cerebrospinal fluid (CSF) or neuroimaging biomarkers. Neuroimaging biomarkers that can identify biological evidence of AD include Aβ positron emission tomography (PET) for brain amyloidosis, tau-PET for NFT pathology, structural magnetic resonance imaging (MRI) for hippocampal atrophy, and fluorodeoxyglucose (FDG) PET for brain metabolic changes [4, 5]. For CSF, three markers (referred to as the core AD biomarkers) can jointly detect “a positive AD profile”. These are: Aβ42 (or Aβ42/Aβ40 ratio), which reflects Aβ plaque pathophysiology; phosphorylated-tau (p-tau), an indicator of tau phosphorylation; and total-tau (t-tau), a neuronal injury or neurodegeneration marker [6, 7]. The concentrations of these biomarkers change in individuals with biological evidence of AD compared with normal controls. Aβ42 is decreased and Aβ40 is unchanged. However, the Aβ42/Aβ40 ratio adjusts for inter-individual differences in the concentrations of the aggregation-prone Aβ42 peptide, making the ratio a more reliable indicator of Aβ plaque pathology compared with Aβ42 alone [8]. P-tau and t-tau levels are both increased in AD versus unaffected controls, with the biomarker concentrations increasing according to disease severity [6, 7]. CSF t-tau is excellent for differentiating AD from healthy controls [5]. CSF neurofilament light (NfL) is another strong indicator of neurodegeneration that can in principle substitute for t-tau in AD, however, unlike t-tau, CSF NfL is also increased in other neurodegenerative diseases [9–11]. Moreover, CSF t-tau, but not CSF NfL, is associated with Aβ pathology in AD [12]. The core CSF biomarkers are reported to be adidtionally changed, in different combinations, in non-neurodegenerative neurological conditions such as traumatic brain injury, Cretuzfeldt Jakob disease, stroke and cardiac arrest [6, 13–16]. For this reason, their specificity to AD should be interpreted in the context of neurodegenerative diseases.

### Shortcomings of current biomarker tools

There is limited availability of cyclotrons for PET radiotracer synthesis worldwide [17]. Similarly, the expertise and resources for CSF biomarker analyses is limited, with a recent study identifying only 40 centers mostly in Europe and North America (and a few in Australia and China) actively involved [18]. Access to, and expertise for, biomarker-supported AD diagnosis and research is therefore acutely limited, excluding most of the global population.

### Blood biomarkers: next-generation AD diagnostics

Blood, being the most ubiquitous biospecimen for clinical chemistry purposes, provides new opportunities to expand access to and participation in AD biomarker research and clinical care [5]. Blood collection procedures do not require specialized training and facilities as lumbar puncture and PET imaging. Furthermore, the costs of blood biomarker analyses are estimated to be a fraction of the fees charged for neuroimaging appointments [19].

The classical AD biomarkers that characterize the disease in the brain and CSF – Aβ, p-tau and t-tau – have also been described in blood (for recent updates see [4, 5, 20, 21]). This review provides a short update on plasma p-tau, the latest addition to the plasma biomarker toolbox. Several new plasma p-tau methods have been described recently from independent academic and pharmaceutical research laboratories that have shown robust technical, clinical and prognostic performances.

### Novel p-tau biomarkers in CSF

In AD context, p-tau biomarkers that work in blood must also have high (if not better) diagnostic and predictive performances in CSF, due to the close contact of the CSF with the brain parenchyma and serving as a sink for brain extracellular solutes [22]. P-tau biomarker performances in CSF have been extremely important to the analytical and clinical validation of plasma P-tau181 as the most widely characterized tau phosphorylation site in CSF, with biomarkers focusing on this epitope currently being used in clinical practice [23–25]. Nonetheless, several other p-tau biomarkers have been described recently. For example, while both CSF p-tau181 and p-tau231 are well-established indicators of ongoing tau pathology, pathological phosphorylation at threonine-231 appears to be observed earlier than at threonine-181 [26]. This observation is useful for biomarker development to detect AD at very early stages prior to symptom onset [12]. Recent studies have also shown that p-tau217 may be more sensitive for familial and sporadic AD than p-tau181 [17, 18]. Nevertheless, the most recent studies showed that standard immunoassays that target phosphorylated tau protein in its mid-region are outperformed by those that capture tau on its N-terminal-to-mid-region peptides/fragments, especially in the preclinical stage [27, 28]. Note that tau is truncated at several defined epitopes [5]. N-terminal-directed p-tau181 and p-tau217 differentiated Aβ + AD dementia from control groups with much greater accuracy and fold-changes than mid-p-tau181 [27, 28]. Moreover, fold changes in AD versus control groups were highest for p-tau217, suggesting superior dynamic ranges over the aforementioned epitopes [28–31]. Nonetheless, p-tau231 shows the strongest topographical associations with the earliest changes in Aβ-PET uptake ahead of p-tau217 and p-tau181 [26, 32], in agreement with neuropathological evidence [33, 34]. More recently, the novel biomarker p-tau235 which becomes abnormal mostly in those already positive for p-tau231 has been described as a potential staging biomarker [35]. Moreover, an assay for tau truncated at amino acid 368 shows strong correlation with tau-PET [36], while the concentration of tau species truncated at 224 also increases according to neuropathological staging [37]. CSF tau fragments starting from amino acid 243 are also shown to associate with tau PET, and could thus be a marker of soluble tau aggregates [38]. Furthermore, brain-derived tau, an assay capturing central nervous system tau released into blood, demonstrates specificity to AD and might reflect neurodegeneration due to AD [39].

The foregoing discussion shows that CSF p-tau biomarkers have proven highly beneficial for the prognosis, diagnosis and staging of AD. However, the limitations highlighted above for CSF markers apply to them as well, making the transition to blood-based p-tau markers much more desirable. It is important to note that it is incorrect to refer to the different p-tau forms or epitopes as "isoforms" as done in some recent publications. This is because isoforms indicate splice variants of a gene, and not the phosphorylation sites in the resulting protein.

## Diagnostic performances of plasma p-tau

**Fig. 1:**
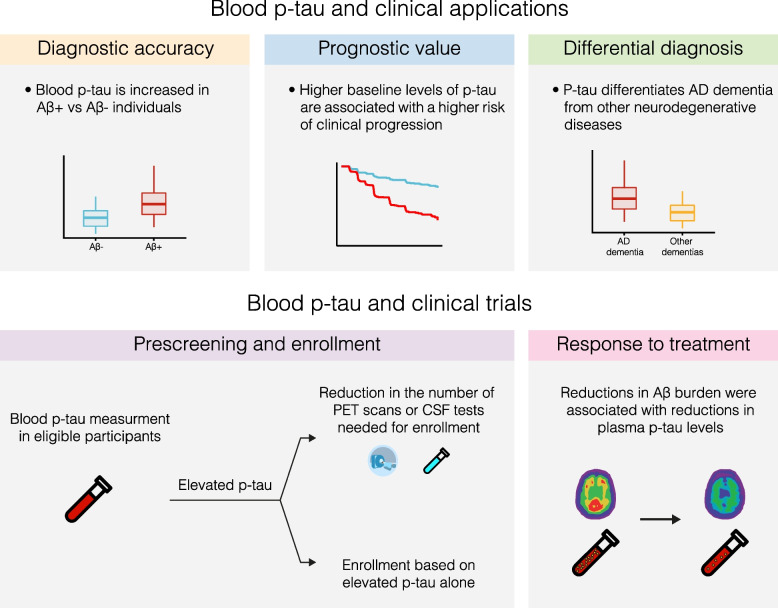
Potential applications of plasma p-tau in clinical care and in therapeutic trials

In this section, we discuss the diagnostic and pathophysiological performances of plasma p-tau, and their associations with Aβ, tau and neurodegenerative pathological changes (Fig. 1).

### Time course of plasma p-tau changes in normal aging and across the AD continuum

Plasma p-tau181, 217 and 231 levels have age associations, although not as strong as those reported for other markers like NfL [32, 40–42]. Young adults (~ 20–30 years of age) have lower concentrations of these markers compared with CU older adults without biomarker evidence of disease [32, 41, 42].

The levels of plasma p-tau181, p-tau217 and p-tau231 each increase with disease severity and the intensity of Aβ and tau pathologies, with higher rates of change for those with abnormal baseline p-tau concentrations [19, 32, 35, 43–49]. When analyzed according to diagnostic groups, these increases tend to plateau in individuals in the late AD dementia stage presumably due to extensive degeneration, resulting in reduced or lost association with CSF and PET biomarkers [19, 41, 43, 45, 48, 50]. A recent study showed that contrary to plasma p-tau181 and p-tau231, p-tau217 demonstrated longitudinal increase in Aβ+ compared with Aβ- individuals, making it a candidate monitoring marker in therapeutic trials [49].

### Plasma p-tau levels in individuals with genetic predisposition to AD and other tauopathies

Although the vast majority (> 90%) of AD patients show sporadic/late-onset forms of the disease (despite strong associations with genetic risks such as *APOE* e4 carriership) individuals with known genetic predispositions present with familial AD [51].

In familial AD, plasma p-tau levels showed increases in pre-symptomatic individuals over a decade before symptom onset [42, 52]. In *APP* and *PSEN1* mutation carriers, plasma p-tau181 and p-tau217 were increased in presymptomatic and symptomatic cases compared with non-carrier controls [42, 53]. Plasma p-tau217 was significantly increased approximately 20 years before the estimated year of onset of MCI while plasma p-tau181 was increased 16 years before the onset of cognitive impairment (in combined MCI and AD dementia cases) [42, 53]. In a study that directly compared plasmap-tau181 and p-tau217 in familial AD participants, p-tau181 only modestly discriminated symptomatic from presymptomatic and was only evident when compared to non-carriers [42]. Plasma p-tau217, on the other hand, differentiated biologically-defined AD from patients without diagnostic levels of AD histology [42].

In adults with Down syndrome (which can be characterized by triplication of the *APP* gene), plasma p-tau181 and p-tau217 discriminated asymptomatic individuals from each of the prodromal and dementia groups [54, 55]. Since a large proportion of people with Down syndrome develop AD symptomatology and pathology during their lives, evaluating biomarker changes in these individuals provides key insights into the biological progression and staging that is important for understanding same in sporadic cases.

To the contrary, in participants carrying mutations in the *MAPT* gene that are known to cause tauopathies other than AD, blood-based p-tau181 levels remained normal as in healthy controls, and in the case of specific mutations the concentrations appeared to be further decreased compared with normal controls [56]. Increased levels of CSF p-tau217 have also been found in non-AD tauopathy carriers of the *MAPT* mutation R406W [57].

### Plasma p-tau associations with clinical and biological evidence of AD and normal aging

Plasma p-tau forms correlate with cognitive capacity assessed with a range of instruments including the Mini-Mental State Examination, the Montreal Cognitive Assessment and the Clinical Dementia Rating-Sum of Boxes (CDR-SOB) [19, 27, 32, 41–43, 45, 48, 58–62]. Baseline plasma p-tau concentrations predict future cognitive decline and progression to MCI and dementia, with performances sometimes paralleling those of CSF p-tau [19, 43, 46–48, 58, 63]. Increased levels of plasma p-tau associate with more rapid decline in cognition, cortical thickness, hippocampal atrophy and glucose metabolism [19, 32, 41, 44, 46, 64–67]. More recently, a comparative study that evaluated p-tau181, p-tau231, and p-tau-217 in a head-to-head manner demonstrated that p-tau217 quantified by IP-MS technology discriminated with higher accuracy patients with MCI and those who progressed to AD dementia [68].

Plasma p-tau levels significantly associated with CSF Aβ42/Aβ40 as well as with Aβ-PET accumulation in early accumulating brain regions (e.g., precuneus, temporal and superior-frontal areas) in preclinical stages, which became stronger and extended to late-accumulating regions (e.g., subcortical structures) later in the disease course [19, 27, 32, 41–43, 45, 48, 53, 58, 59, 61, 67–69]. In neuropathology studies, similar positive associations were recorded against various Aβ staining methods such as Thal, CERAD, and thioflavin stain scores [32, 35, 42, 45, 46, 70]. Furthermore, plasma p-tau concentrations associated with tau biomarkers (i.e., NFT pathology at postmortem, CSF p-tau or tau-PET) in the AT(N) framework [19, 27, 32, 41–43, 45, 46, 48, 50, 59–62]. Plasma p-tau also associated with brain atrophy, FDG PET, CSF t-tau or CSF NfL [19, 27, 32, 41–43, 45, 46, 48, 60–62]. In Down syndrome, plasma p-tau181 correlated with atrophy and hypometabolism in temporoparietal regions [54]. When more than one p-tau form was included in a study, plasma p-tau217 generally showed stronger associations with brain Aβ deposition than p-tau181 and p-tau231 [49, 62, 68, 69, 71]. Moreover, the IP-MS plasma p-tau217 method performed better than immunoassay-based ones in a recent comparative study [68].

### Head-to-head comparisons of plasma p-tau forms

Recent studies comparing the performances of plasma p-tau217 and/or p-tau231 with p-tau181 assays from different academic and industrial sources have shown that they have equally robust analytical performances and diagnostic capacities to identify individuals with AD pathology versus biomarker-negative normal controls or non-AD tauopathies (except plasma p-tau231 from ADx NeuroSciences which may need further improvement) [32, 58, 62, 70, 72] signifying that these biomarkers are ready for widespread clinical and research use. Plasma p-tau concentrations increase gradually along the sporadic AD continuum in relation to the severity of Aβ pathology and cognitive function, reaching the highest concentrations in Aβ + participants with MCI and AD dementia [19, 41, 43–45, 47, 58, 62, 72, 73]. Plasma p-tau181, p-tau217 and p-tau231 each differentiates between Aβ- CU individuals versus Aβ + CU (preclinical AD), Aβ + MCI, and Aβ + AD dementia with good accuracies, while improving clinical characterization of cognitive performance [19, 27, 32, 41–43, 45, 46, 48–50, 58–62, 68, 69, 71–82].

The largest fold increases (compared with Aβ- CU) are observed for plasma p-tau217, followed by p-tau231 and p-tau181 [21, 32, 61, 62, 68, 72, 83] in agreement with CSF data [26–29]. To this end, p-tau217 is the most analytically challenging of the p-tau biomarkers to measure since the levels are very low in those without (e.g., Aβ- CU and Aβ- non-AD dementias) and those with emerging Aβ pathology (including preclinical stages) [72].

From research perspectives, however, plasma p-tau217 and p-tau231 each tends to show earlier and stronger associations with Aβ and tau pathologies than p-tau181 [32, 42, 70, 71, 78, 84], including correlating with Aβ accumulation in early brain regions and with tau pathology in MCI patients with temporal lobe pathology [32, 35, 42, 70]. Ashton et al*.* (2021) showed that plasma p-tau231 is a promising biomarker in AD due to its diagnostic accuracy in early stages, and its association with incremental levels of brain Aβ pathology even before abnormality thresholds of Aβ-PET are reached [32]. Plasma p-tau231 was superior to both plasma p-tau181 and CSF p-217 for this purpose [32]. Moreover, plasma p-tau217 is a promising candidate biomarker for AD. p-tau217 appears earlier and has a stronger association with AD pathology than plasma p-tau181 in preclinical AD [42, 70, 85]. Recent data support these arguments, and further demonstrated that p-tau231 is the first to increase in preclinical AD (A + T-) [71]. However, p-tau217 becomes abnormal shortly after (at the A + T + stage), following which this biomarker shows faster longitudinal increases compared with p-tau231. Plasma p-tau181 also becomes abnormal in A + T + individuals but with less robust longitudinal change versus p-tau217. Therefore, p-tau181 seems to be mostly associated with changes corresponding to widespread amyloidosis. These findings also explain why plasma p-tau181, p-tau217 and p-tau231 all have excellent diagnostic performances for symptomatic AD but p-tau217 and p-tau231 have improved accuracies at the preclinical stages. Together, these finding support the use of specific plasma p-tau biomarkers for staging and tracking AD progression.

However, all these plasma p-tau forms become abnormal ahead of tau-PET, suggesting that they can predict the outcome of PET imaging [19, 32, 41, 43, 48, 85]. In line with this, high levels of plasma p-tau are present even in preclinical stages of AD and can predict changes in tau-PET [19, 32, 41, 43, 48, 85].

Recent studies suggest that longitudinal levels of plasma p-tau217 could reflect the relation between amyloid pathology and tau deposits [44, 70] which would make it a suitable biomarker for both amyloid and tau pathologies disease progression.

Although plasma p-tau is mostly validated in cohorts of individuals pre-classified according to PET or CSF biomarker results, a few studies in population-based cohorts categorized solely by clinical diagnosis give a glimpse into potential uses as a pre-screening tool. For example, Simrén et al. [47] showed that plasma p-tau181 is increased in a subset of individuals at the MCI and AD dementia stages, and correlate with cognitive impairment and gray matter atrophy. In individuals presenting to the primary-care clinic with suspected cognitive decline and given preliminary diagnosis without biomarker testing, plasma p-tau181 and p-tau231 discriminated those with cognitive impairment from normal controls, however the biomarkers were unable to differentiate between those given preliminary diagnoses of MCI or AD [32, 41].

### The value of plasma p-tau to differentiate AD from other neurodegenerative diseases

Plasma p-tau181, p-tau217 and p-tau231 each distinguished AD from non-AD tauopathies such as frontotemporal dementia, progressive supranuclear palsy and corticobasal degeneration [32, 41, 42, 45, 48, 56, 60, 61, 86]. In studies with postmortem validation, the discriminatory accuracies between Aβ + AD and Aβ- non-AD cases were as high as > 90%, with plasma p-tau being able to further distinguish between non-AD cases with or without concomitant AD pathology [32, 42, 45, 46, 48, 60, 61, 86].

Separating cognitive impairment due to AD versus dementia with Lewy bodies (DLB) is difficult to establish clinically because up to 50% of DLB patients are also thought to have concomitant AD [87]. Plasma p-tau181 levels differentiated between autopsy confirmed AD and DLB, and went on to show that DLB patients with AD co-pathology have higher p-tau concentrations than those without [45]. In DLB patients with a positive CSF Aβ profile, plasma p-tau181 and 231 levels were higher than those of normal controls and DLB participants with a negative Aβ profile but lower than those of AD patients, correlating with cognitive performance [67]. Similarly, plasma p-tau181 and p-tau217 correlated with CSF biomarkers, Aβ PET and tau PET in clinically-diagnosed DLB patients to suggest that these biomarkers have capacity to identify AD co-pathology in DLB [88].

### Plasma p-tau versus other biomarkers

Plasma p-tau181, p-tau217 and p-tau231 individually performed significantly better than the diagnostic capacities of each of *APOE* ε4 carriership, plasma NfL, t-tau, and the Simoa Aβ42/Aβ40 [32, 41, 42, 50]. When compared against non-phospho-tau blood biomarkers – NFL, Aβ ratio, t-tau and glial fibrillary acidic protein – plasma p-tau were significantly better at differentiating between AD and CU individuals [89]. These results were comparable to those of predictive models incorporating Aβ PET, age, sex and *APOE* ε4 carriership [59].

### Diversity in plasma p-tau cohort validation studies

Plasma p-tau studies have so far been performed in research cohorts in Europe and North America, with a few studies form Australia and Asia. The included volunteers in most studies identified as non-Hispanic Whites, and were also mostly of high socio-economic status (e.g., highly-educated, high-earning jobs, communities with high neighborhoods index). On the other hand, people living in other neighborhoods and those of other socioeconomic statuses are yet to be studied. Moreover, racial and ethnic diversity in research participation has been minimal. At the time of writing this manuscript, only three studies have included significant numbers of ethnoracially diverse participants [70, 81, 90]: one investigated plasma p-tau181 in relation to amyloid accumulation and AD diagnosis in a Singaporean cohort of high baseline cerebrovascular burden [81] while another probed plasma p-tau217 and p-tau181 in a multi-ethnic, community based cohort in the United States [70]. Furthermore, Schindler et al. [90] studying non-Hispanic White and African-American pairs of older adults of the same demographic characteristics (age, sex, cognition and *APOE* ε4 genotype) recently demonstrated that the predictive accuracies of plasma p-tau231 and p-tau181 identify abnormal Aβ-PET and CSF Aβ42/Aβ40 results significantly differ in the participants who represented the two racial groupings studied.

Another point worth discussing is that most cohorts evaluated so far have been from memory clinics or are clinical research cohorts; population-based studies are missing [5]. A recent study of community-dwelling older adults in a socioeconomically deprived region of southern Pennsylvania showed that plasma p-tau181 (the only p-tau marker assessed) levels were significantly higher in those with compared with those without cognitive impairment [91].

Another important factor that should addressed is the effect of comorbidities; Mielke et al. [40] found that chronic kidney disease associates with plasma p-tau181 and p-tau217 levels with a similar effect size as that between Aβ + and Aβ- individuals.

## The path to diagnosis and therapeutic trial applications

Plasma p-tau biomarkers can, as highlighted above, capture relevant clinico-biological information in AD, with the advantages of less invasive collection and cost-effectiveness in comparison to established CSF and PET biomarkers. These factors, alongside the AD-specific characteristics (in comparison to other biomarker such as plasma NfL [46, 49, 82]) and analytical advantages (in comparison to plasma Aβ42/Aβ40, which presents challenges due to low disease-related fold changes and narrow analytical detection range [5, 92]), make plasma p-tau biomarkers more scalable candidates for implementation. This newly-achieved technical feasibility of large-scale in vivo detection of AD has several implications for clinical trials, epidemiologic research and public health (Fig. 1).

### Clinical diagnosis and prognosis

Plasma p-tau has vast potential to support AD diagnosis and prognosis (Fig. 1). We propose that these biomarkers are integrated into the existing diagnostic workup at both primary and specialist care hospitals. In the primary care setting, plasma p-tau could be used to pre-screen for AD pathophysiology. When combined with the regular clinical workflow for suspected dementia, altered levels of plasma p-tau in patients with cognitive symptoms would point to potential AD (or at least AD-associated amyloidosis) while those with normal concentrations are further evaluated for non-AD causes of cognitive symptoms. In patients whose clinical profiles fit AD (e.g., those with family history of the disease and/or have confirmed genetic predisposition for AD) but have their plasma p-tau in normal ranges, periodic follow-up clinical and blood biomarker assessments (e.g., annually) would be ideal to monitor for longitudinal changes in p-tau and cognitive capacity.

All patients showing increased plasma p-tau levels at the primary care clinic should be referred to secondary care for their plasma biomarker results to be compared with more extensive dementia assessment outcomes and, if necessary, confirmed by CSF or PET. Similarly, those with symptoms suspected to be due to non-AD causes would also be verified to be without biomarker evidence of AD by either CSF or PET *ATN* biomarkers. In patients whose plasma p-tau profiles are confirmed at the specialist clinic, the blood biomarkers would be further useful to follow disease progression over several years. As the continue to learn more about blood biomarkers and their analytical robustness and diagnostic accuracies improve, it is feasible to envisage that the need to confirm results with CSF biomarker measures will reduce over time. A future of standalone blood biomarker evaluations may not be too far away.

### Clinical trials

The development of clinically effective disease-modifying therapies remains a challenge. Some anti-Aβ immunotherapy candidates, have demonstrated to be biologically effective in clearing amyloid from the brain [93], while failing to robustly meet pre-specified cognitive endpoints [94]. In 2021, the anti-Aβ drug aducanumab was approved by the United States Food and Drug Administration based on the results of two parallel phase-3 trials, ENGAGE and EMERGE, that had been previously interrupted in futility analyses. However, post-hoc analyses on the group of participants that completed the study revealed that EMERGE had achieved its primary and secondary endpoints, while ENGAGE did not, with both of them showing amyloid-related imaging abnormalities as a prevalent side effects [95]. This has generated much debate, since many consider that the statistically significant findings from EMERGE may not be of high clinical relevance [96]. Moreover, other anti-Aβ drugs also demonstrated similar or better performance in comparison to aducanumab, such as the phase 2 donanemab trial, which achieved its primary endpoint on slowing cognitive decline as measured by the Integrated Alzheimer’s Disease Rating Scale [97]. More recently, phase III trial of the Aβ aggregate-targeting experimental drug lecanemab met its primary endpoint of significantly reducing cognitive decline and reducing markers of brain Aβ deposition in a large multi-center evaluation of early AD, which was approved by the FDA [98]. Plasma biomarker results are expected to follow soon. With the field rapidly moving towards a treatment response phase, understanding how blood biomarkers can be incorporated into the drug development pipeline is highly needed, given their potential to be used in pre-screening and in monitoring treatment response and safety.

### The role of plasma p-tau in trial enrolment

With the development of biomarkers and advances in diagnostic guidelines, the understanding of AD as a clinico-biological entity has directly impacted trial design, with new clinical studies progressively adopting biomarker-evidence of AD as enrollment criteria. Usually, these trials screen eligible participants with PET or CSF biomarkers and then randomize only those participants with abnormal biomarker profiles according to established thresholds. Considering trials evaluating anti-Aβ and anti-tau therapies need to assess target engagement throughout the study, PET measures are often preferred as the enrollment biomarker. In this context, plasma p-tau biomarkers may not have the same hierarchical status as CSF and PET, but as they associate with and predict PET results and are relatively inexpensive, accessible and less invasive, they are the ideal tools to pre-screen clinical and demographically eligible individuals (Fig. 1). Several strategies have been discussed for this purpose, such as applying plasma p-tau to pre-screen individuals for the presence of Aβ pathology and also to detect eligible participants who are at greater risk of tau accumulation. The plasma p-tau diagnostic accuracy for Aβ positivity has been widely reported in independent studies, and a recent review article suggested that, by adding a plasma p-tau181 to pre-screen for Aβ-PET pathology, up to ~ 60% of the original cost could be saved in comparison to pre-screening only with Aβ-PET, one of the conventional approaches [5, 19]. Regarding Aβ and tau accumulation, Moscoso and colleagues first demonstrated that plasma p-tau181 was associated with longitudinal changes in Aβ-PET in early accumulating regions [43], and then showed that it was capable to identify individuals at higher risk for longitudinal tau accumulation, performing particularly better in cognitively unimpaired individuals with a higher Aβ burden [99], a group of special interest for future pre-symptomatic trials. Similarly, in a recent study by Leuzy et al., the two strongest predictors of tau-PET accumulation were plasma p-tau217 and baseline tau-PET, with the former being the predictor contributing the most in Aβ-positive CU individuals and the latter in Aβ-positive MCIs [100].

Regarding real-life clinical trial applications of such advances, the TRAILBLAZER-2 (Eli Lilly; NCT04437511) donanemab trial for early AD tested the potential of a pre-screening strategy with plasma p-tau181 combined before proceeding to Aβ- and tau-PET [101]. Among the subset of 752 candidate participants who had their plasma p-tau181 levels quantified, 63% of those with elevated p-tau181 had subsequent positive scans for both Aβ- and tau-PET. In contrast, only 37% of the 3619 candidates that had been pre-screened straight away with Aβ- and tau-PET demonstrated positive scans for the two proteinopathies [101]. Based on the success of the plasma pre-screening approach, the same company has taken a step further for their TRAILBLAZER-3 donanemab trial in a large sample of asymptomatic older adults (NCT05026866) [102]. The study is the first to use plasma p-tau (p-tau217) as the sole enrollment criteria. Participants will have their definitive enrolment decision based on plasma p-tau217 levels “consistent with the presence of amyloid and early-tau pathology”, and Aβ-PET is not included in any part of enrollment workflow nor amongst the secondary outcomes [102]. Given that plasma p-tau analytical standardization have not yet been achieved, and the absence of validated strategies for plasma p-tau results interpretation, such a strategy could be susceptible to giving anti-Aβ therapy to asymptomatic individuals without Aβ pathology, a problem that a biomarker-based AD definition had been proposed to resolve [103, 104]. However, the higher performance of p-tau217 (in comparison to p-tau181) and the success from the TRAILBLAZER-2 strategy may indicate potential efficacy for such a bold enrollment criterion. Still, it is important to consider that, unlike the previous trials that focused on early AD dementia, TRAILBLAZER-3 is a prevention trial in asymptomatic individuals, a group that presents mild-to-moderate fold changes in plasma p-tau biomarkers – even for p-tau217 – in Aβ + individuals [42, 61].

In summary, plasma p-tau biomarkers demonstrate great potential to be applied in the clinical trial recruitment flowchart, with clear potential for pre-screening, while results for TRAILBLAZER-3 could be indicative on whether they could be used as a standalone biomarker enrollment criterion.

### Monitoring drug activity

While actual target engagement for the main anti-Aβ and anti-tau trials has been determined by PET measures of the respective target, plasma p-tau biomarkers could offer a minimally-invasive option for monitoring drug activity of new interventions, which is crucial not only for advanced phases but for the whole drug development pipeline (Fig. 1). A blood biomarker capable to monitor drug activity would allow for more frequent time-points in comparison to Aβ-PET, also with the potential of remote sampling, and would also represent, to some extent, what types of treatment response could be seen in the future when the drugs start to be widely applied in clinical practice.

Considering that plasma p-tau associates with both Aβ and tau pathologies [105], in theory it is possible that blood p-tau biomarkers are able to reflect activity of either anti-tau or anti-amyloid therapies. In 2021, the first results evaluating plasma p-tau levels during disease modifying trials were shared with the field. Results from both the ENGAGE and EMERGE aducanumab trials showed that 13–16% reductions in plasma p-tau181 were observed in the high- and low-dose groups in comparison to placebo on treatment week 56 [95, 101]. Moreover, results from the concluded TRAILBLAZER-ALZ donanemab trial, that had more frequent sampling, demonstrated that levels of plasma p-tau217 dropped 24% in comparison to placebo as early as on treatment week 12 [106]. In both cases the changes agreed with reductions in Aβ-PET uptake suggesting that plasma p-tau is associated with brain Aβ accumulation [107, 108]. Interestingly, in TRAILBLAZER-3 the group-level p-tau217 reductions generally persisted even in the subgroup that had discontinued donanemab after 24 weeks due to lack of significant Aβ-PET changes [101]. Nevertheless, it still remains unknown whether plasma p-tau levels would be affected by more effective anti- tau therapies in the clinic. This raises the question of how certain one can be that changes in soluble p-tau are solely due to intervention-mediated removal of Aβ plaques – or potentially associated with yet undetermined clearance of peri-plaque dystrophic neurites containing tau tangles – or if they could be achieved by removing tau tangles from the brain. When such information becomes available, a better understanding on the biological meaning of soluble p-tau will be achieved, since currently it is not entirely possible to disentangle its dual association with AD key neuropathological features.

In brief, these results indicate that plasma p-tau can be a promising biomarker to monitor drug activity of disease modifying treatments in AD. Further trials studies should continue to address their value in treatment response, potentially increase sampling frequency by testing remote collection, and, most importantly, carry detailed analyses of individual-level clinical trial data to determine in which cases reductions in p-tau can identify an effective clinical and biological treatment response.

## Conclusions

Recent breakthrough advances in biochemistry and clinical chemistry have enabled the development of ultrasensitive and robust plasma p-tau biomarkers with the potential to lead the AD field in new directions. Accumulating evidence from multiple independent cohorts using different plasma p-tau assays show that these biomarkers have shown excellent diagnostic accuracies as well as performances that demonstrate capacity to predict post-mortem diagnosis and the outcomes of CSF and neuroimaging biomarker testing. While plasma p-tau181, p-tau231 and p-tau217 have all shown excellent diagnostic utility for the symptomatic stages of AD, plasma p-tau217 and p-tau231 have emerged as markers of incipient AD that become abnormal earlier ahead of p-tau181, especially in the preclinical phase. Since these biomarkers associate to different degrees with amyloid and tau pathology at various stages of the AD continuum, we find it plausible that different p-tau biomarkers will be more suitable for various purposes, especially to evaluate preclinical disease. However, in the case of detecting symptomatic AD, all p-tau biomarkers perform equally well.

Together, these findings show that it is prime time that plasma p-tau biomarkers were employed to support clinical diagnosis as well as to recruit volunteers for therapeutic trials and to monitor the efficacy of drug interventions. In clinical diagnosis, abnormal levels of plasma p-tau would signal a high probability of AD pathophysiology underlying cognitive decline. This observation would be strengthened if plasma NfL are in normal ranges. In clinical trials, pre-screening potential volunteers with plasma p-tau would enrich the population of individuals with high likelihood of AD who could then receive CSF or PET assessments for confirmation (Fig. 1).

### Outstanding questions

As the field moves towards widespread clinical and research implementation of blood biomarkers, it is important to identify and mitigate against physiological and lifestyle factors that can inadvertently introduce measurement errors independent of analytical procedures. As biomarker availability and accessibility increase, so will repeated sampling for clinical assessments and longitudinal evaluations become more common. It is absolutely essential to differentiate between biomarker changes due to pathological and treatment effects from variability induced by physiological and lifestyle factors. Future research should establish if everyday factors like sleep, circadian rhythm, exercise, medical comorbidities, fasting and diet affect the reproducibility of blood biomarker measurements. The results will be important to identify potential sources of error, addressing which should minimize false positivity and false negativity. Furthermore, the results will be critical to developing evidence-backed pre-analytical guidelines for blood handling. Standardization and harmonization of plasma p-tau results collected from different centers and in using different assays will be essential for cross-cohort comparison of results and the generation and validation of cut-points.

Moreover, plasma p-tau must be validated in a broad range of populations that reflects the diversity of the larger community in which these blood biomarkers will be applied. This includes people of different socio-economic statuses, ethno-racial identities, age, cognitive functions, as well as those living in various countries.

## Data Availability

All the data are included in the manuscript.

## Abbreviations

AD: Alzheimer’s disease
p-tau: Aβ amyloid-beta
CSF: Cerebrospinal fluid
CU: Cognitively unimpaired
CI: Cognitively impaired
DLB: Dementia with Lewy bodies
MCI: Mild cognitive impairment
MRI: Magnetic resonance imaging
NfL: Neurofilament light chain
NFT: Neurofibrillary tangles
PET: Positron emission tomography p-tau, phosphorylated tau
t-tau: Total-tau
